# The Relationship of Molluscum Sebaceum (Keratoacanthoma) to Spontaneously Healing Epithelioma of the Skin

**DOI:** 10.1038/bjc.1953.6

**Published:** 1953-03

**Authors:** F. A. Fouracres, J. W. Whittick

## Abstract

**Images:**


### THE RELATIONSHIP OF MOLLUSCUM SEBACEUM (KERATO-ACANTHOMA) TO SPONTANEOUSLY HEALING EPITHELIOMA OF THE SKIN.

F. A. FOURACRES* AND J. W. WHITTICK.

*From the Department of Pathology, Royal Cancer Hospital, London.*

Received for publication December 13, 1952.

THERE is an astonishing neglect in the literature of the benign cutaneous condition described by MacCormac and Scarff in 1936, and named by them molluscum sebaceum. An alternative name, kerato-acanthoma, has since been applied, and this has the merit of preventing confusion with molluscum contagiosum, an unrelated condition. It has been the subject of only 4 papers, all by British authors, MacCormac and Scarff (1936), Musso and Gordon (1950), Rook and Whimster (1950) and Beare (1953), and does not yet appear to have reached text-books of dermatology or pathology. That this in no way reflects the incidence of molluscum sebaceum is certain. Indeed, it is not an uncommon condition, and is still very frequently misdiagnosed as squamous-cell carcinoma by both dermatologist and pathologist.

Smith, in 1934, described the occurrence in a young man of multiple primary squamous-cell carcinomas of the skin which healed spontaneously and he was unable to find any comparable case in the literature. Since that time there have been eight papers on the subject (Table II) with descriptions of similar lesions in 11 patients. Comparison of the clinical and pathological descriptions of these cases with the natural history and the structure of molluscum sebaceum at once suggests the probability that all the recorded cases of spontaneously healing epithelioma of the skin are in fact instances of molluscum sebaceum.

The following account is based on a series of 7 cases of molluscum sebaceum which after biopsy diagnosis were untreated and allowed to run their natural course to spontaneous regression. Numerous other cases have been studied, but are not included here because they were treated by radiotherapy or surgery.

### *Molluscum sebaceum.*

*Clinical features.*—Some of the clinical features of the lesions in our 7 cases are summarized in Table I.

*Histology.*—The histological structure of molluscum sebaceum was admirably described and illustrated by Dunn and Smith (1934), though, unfortunately, under the diagnosis of " Self-healing primary squamous carcinoma of the skin." That they were not fully convinced of the correctness of the diagnosis is suggested by their statement that " It may be we are dealing with something which is not quite cancer in the ordinary accepted sense, but which cannot in its early stages be

* Medical Research Council Scholar.

TABLE I.—*Cases of Molluscum Sebaceum Untreated after Biopsy Diagnosis.*

| Case. | Age. | Sex. | Occupation. | Site of lesion. | Period of active growth prior to first attendance (weeks). | Total duration of lesion (weeks). | Final result. |
|---|---|---|---|---|---|---|---|
| I | 50 | M. | Mill foreman | Neck | 3 | 18 | Small white scar. |
| II | 54 | M. | Toolkeeper | Chest | 3 | 15 | White scar. |
| III | 49 | M. | Foreman plater | Cheek | 4 | 16 | Small pitted scar. |
| IV | 50 | M. | Labourer | Eyelid | 2 | 7 | Small scar. |
| V | 54 | M. | ,, | Cheek | 2 | 8 | Small depressed scar. |
| VI | 51 | M. | Foreman process worker | ,, | 2 | 9 | Pitted scar. |
| VII | 69 | F. | Household worker | Face | 6 | 14 | No scar. |

differentiated from cancer, either histologically or clinically." With the present knowledge of molluscum sebaceum, experience and adequate biopsy material it is possible now in a large percentage of cases to separate molluscum sebaceum from squamous-cell carcinoma.

As Rook and Whimster (1950) have described the histological changes during growth and regression of molluscum sebaceum, only the fully developed lesion will be described here. Of this stage, the structure illustrated (Fig. 2) is fully typical. In a central vertical section it is seen as an invagination or crypt containing keratin and lined by hyperplastic epidermis, which internally forms papillary projections and externally penetrates the dermis as cords and cell nests. Through a central opening, of variable size, the keratin projects on the skin surface. The surrounding epidermis, as it extends over the dome-shaped lesion, becomes attenuated and loses its rete processes. At the margin of the central pore the epithelium is acutely reflected and, inverted, runs parallel to the skin surface for a variable distance before curving to form the lateral walls and then the floor of the central crypt. Keratinization within the hyperplastic stratified squamous epithelium forming the walls of the central cavity leads to the formation of secondary, keratin-filled crypts which discharge into the main cavity (Fig. 3). Their walls form irregular, papilliferous projections into the main crypt. From the deep aspect of the hyperplastic epithelium, irregular, keratinizing cords and cells nest (Fig. 3) of progressively and rapidly diminishing size invade the surrounding dermis, destroying and engulfiing connective-tissue fibres (Fig. 4) and arrector pili muscles. At the advancing margin (Fig. 5), variation in nuclear size and intensity of staining may be pronounced, and mitoses are fairly numerous while the lesion is enlarging. In contrast to this appearance of active invasive growth are the rapidity and completeness of differentiation in the hyperplastic epithelium, which may in places even develop a stratum granulosum.

In most cases, the deep margin remains well within the dermis but occasionally, with secondary infection, penetration may be deeper. Hair follicles and sweat ducts may be engulfed in the hyperplastic epidermis and the ducts frequently show squamous metaplasia of their lining epithelium.

About the lesion there is infiltration of the dermis by leucocytes—lymphocytes, plasma cells and polymorphonuclears—amongst which eosinophilic polymorphs may be prominent. Foreign-body giant cells, grouped about keratin, may be present at this stage, but are more commonly found during the stage of regression. No inclusion bodies are present.

That the lesion of molluscum sebaceum closely simulates highly differentiated squamous carcinoma will be apparent from the histological description and illustrations. There is no single feature which will allow differentiation. With a biopsy specimen adequate in size and depth, however, a histologist familiar with the picture of molluscum sebaceum will, in most cases, be able to make a correct diagnosis. It has about it an appearance of benignity which is difficult to define but which is due probably to its regularity of structure, relatively superficial position in relation to its size and the completeness of differentiation of the hyperplastic epithelium. The clinical appearance (Fig. 1(*a*)) and a history of rapid growth over a period of 4 to 8 weeks are essential aids in diagnosis.

### *Spontaneously Healing Epithelioma.*

*Clinical features.*—Table II summarizes some of the clinical features of all cases described as spontaneously healing epithelioma.

*Histology.*—The histological picture in all recorded cases of spontaneously healing epithelioma is identical with that of molluscum sebaceum.

### DISCUSSION.

The recorded cases of multiple spontaneously healing epithelioma possess characteristics closely similar to those of molluscum sebaceum, whilst differing markedly from ordinary squamous carcinoma, single or multiple. These features will be described from the following points of view : rate and duration of growth, local and lymphatic spread, multiplicity and seat of lesions.

### *Rate and duration of growth.*

The lesions of both molluscum sebaceum and spontaneously healing epithelioma have a short period of very rapid growth, lasting 4 to 8 weeks, followed by a stationary period and then regression to complete resolution. In untreated cases the whole process is complete in 4 to 6 months. Variations in size, duration and extent of scarring occur with secondary infection. Not only is their rate of growth and course the same, but their clinical appearance during these stages is identical. Each begins as a minute papule, which rapidly enlarges to become, at the end of the period of growth, a circular or oval umbilicated nodule of 1 to 2 cm. in diameter, which is raised $\frac{1}{2}$ to 1 cm. above the skin surface and has a keratotic centre (Fig. 1 (*a*)). About this central plug of keratin is a broad, dull red, convex and smooth-surfaced epidermal rim. With regression there is gradual flattening of the lesion, and finally the keratin plug falls off to leave a clean, slightly depressed scar.

Ordinary squamous carcinoma usually has a much slower rate of growth with progressive increase in size for several months. Although rapid growth may occur, it is usually accompanied by early involvement of the regional lymph glands. That rapidity of growth has not been accompanied by metastasis to the local lymph nodes in the spontaneously regressing cases has indeed been stressed by some of the authors. Dunn and Smith (1934) state of the lesion in their case, " It had already in the short time of 3 to 5 weeks reached a size which squamous epithelioma usually takes as many months to attain. Rapidity of growth is usually associated with early involvement of the regional glands, but this was not the case here."

TABLE II.—*Recorded Cases of Spontaneously Healing Epithelioma.*

| Case. | Author. | Age. | Sex. | Age at onset. | Occupation. | Family history | Site of lesions. | Approximate duration of individual lesions (months). |
|---|---|---|---|---|---|---|---|---|
| 1 | Smith (1934) | 23 | M. | 16 | Miner | — | Face, ears, arms, thighs, legs | 6 |
| 2 | Dunn and Smith (1934) | 42 | M. | 42 | Sheet-iron worker | — | Thigh, leg. | — |
| 3 | Smith (1948) | 58 | M. | 29 | Coal miner | ? Father | Face, ears, nose, neck, arms, legs | 3 |
| 4 | Ayres (1948) | 63 | M. | 40 | — | — | Face, ears, hand | — |
| 5 | Grzybowski (1950) | 57 | M. | 49 | Motor mechanic | — | Scalp, face, ears, neck, upper thorax | 4 to 8 |
| 6 | Sommerville and Milne (1950) | 34 | F. | 18 | Member of Armed Forces | Mother, brother, maternal aunt | Scalp, face, ears, nose, fore-arms, legs | 4 |
| 7 | Charteris (1951) | 69 | M. | 23 | — | ? Father | Face, forearms, perineum | 4 to 8 |
| 8 | „ „ | 28 | M. | Before 21 | Apprentice printer | Son of Case 7 | Face, ears, thigh, leg | — |
| 9 | „ „ | 43 | M. | 38 | Ditto | „ „ | Face, ears, wrist, leg | — |
| 10 | „ „ | 29 | M. | 13 | Apprentice engineer | „ „ | Face, ears | — |
| 11 | Witten and Zak (1952) | 49 | M. | 48 | Leather inspector | — | Whole body—face, neck, trunk, limbs | — |

*Local and lymphatic spread.*

Were the alleged cases of spontaneously healing epithelioma true instances of squamous carcinoma there should be good evidence of local destructive invasion and lymphatic extension, but this is lacking. In over half the recorded cases the regional glands are not mentioned and, in view of the long survival period in these cases, it may reasonably be presumed that they were not involved. For example in Smith's (1948) Case 1 there is a history of lesions for 21 years and in his Case 2 for 29 years. The patient of Sommerville and Milne (1950) had multiple lesions over a period of 22 years. Lack of lymphatic involvement was mentioned by Dunn and Smith (1934), and Grzybowski (1950) comments on " the lack of involvement of lymph nodes in spite of the 9 years' duration of the disease." In only one case, Charteris' second (Charteris, 1951), is enlargement of lymphatic glands mentioned, and these were found, histologically, to be tumour-free.

Local invasion, too, appears to have been very limited in the spontaneously healing cases, being restricted to local penetration into the dermis. Thus, Dunn and Smith (1934), " the invasive process at its height never appears to transgress the limits of the true skin." This feature applies equally well to molluscum sebaceum. Description by two authors of local tissue destruction in spontaneously healing epithelioma requires comment. Smith (1948), when recording the subsequent history of his Case 1, states that he was an irregular attender and " permitted much further destruction of the soft tissues of his face," and that he now wore a prosthesis to cover extensive destruction of his nose. This on first reading seems proof of an infiltrating and destructive malignant growth, but it must be pointed out that in the previous paragraph we are told that following treatment of a lesion of the right leg by radium, the same patient had to undergo amputation of his leg because of necrosis of the tibia. Lesions of his face had been treated by contact X-ray therapy. If this extensive destruction of the nose, requiring a prosthesis, was indeed due to squamous carcinoma, then the lack of lymphatic extension and the ultimate spontaneous healing (*sic*) make this case even more remarkable. The second author, Charteris (1951), gives the final reference to his first patient as " eventually we found that he died in November, 1945, from rapid and extensive recurrence of the anal lesion." But the last contact with the patient had been in April, 1945, when he attended hospital presumably with no recurrence of his anal lesion, and of the terminal condition there is neither biopsy nor necropsy confirmation.

*Multiplicity and seat of lesions.*

In all 11 recorded cases of spontaneously healing epithelioma the lesions were multiple. There was either progressive generalised extension from an initial lesion or sporadic eruption of a few lesions over many years. This feature of multiplicity seems contradictory to spontaneous healing of a malignant growth, for it is reasonable to expect that a person with such a remarkable power of control over a malignant process should have some resistance to the formation of similar malignant tumours. It is of interest that the converse holds, namely that there is no report of spontaneous healing of a solitary squamous carcinoma. That spontaneous healing should be confined to multiple primary squamous carcinoma seems contrary to the laws of probability.

It is well known that primary squamous carcinoma may be multiple, but these develop in skin which has previously undergone some pathological, " precanccerous " change such as senile keratosis or dermatitis due to X-rays or arsenic. The spontaneously healing epitheliomas, on the other hand, have all developed in healthy skin. To quote Smith (1934), " photographs show how normal and ' young ' the skin is, apart from the actual lesions and scars." They have displayed, therefore, not only the rarity of multiplicity itself but of multiplicity without " precancerous " lesions, and also despite the ability of the host to produce spontaneous healing.

If, as appears possible, an infective agent is the cause of molluscum sebaceum, the bodily distribution and the familial incidence in cases of spontaneously healing epithelioma might readily be explained. In both Grzybowski's (1950) and Witten's and Zak's (1952) patients the progressive spread from a lesion on the scalp to the face, ears, neck, chest and trunk is very suggestive of auto-infection. Another interesting feature in this connection is the rapid spread of the lesions under bad hygienic conditions. While 3 of these patients were prisoners of war there was an enormous increase in number of their lesions. With regard to the familial incidence noticed in 3 of the 11 patients, the following members of each family had similar lesions :

1. Somerville and Milne (1950) : the patient's mother, brother and maternal aunt.
2. Charteris (1951) : the patient's father and three sons.
3. Smith (1948) : Case 2 : the patient's father.

This has been stated to be due to a familial inherited tendency towards squamous carcinoma of the skin. Yet what would appear to be a directly opposed factor—ability of the same individual to produce spontaneous healing—is also ascribed to an inherited factor. However, if all the cases described as spontaneously healing epithelioma were in fact molluscum sebaceum the explanation is much simpler, and there is no need to search for an inherited diathesis. Infectivity would account for spread within a family and spontaneous healing would be the natural termination.

### SUMMARY.

Attention is drawn to the close similarity, in structure and natural history, between molluscum sebaceum and the cutaneous lesions described in the literature as multiple spontaneously healing epithelioma. It appears probable that the two conditions are identical, and that there is no acceptable record of spontaneous regression of a squamous carcinoma of the skin.

Dr. Hugh Gordon has very kindly allowed us to make use of the clinical records of the patients with molluscum sebaceum. The photomicrographs are by Mr. L. A. Cowles from histological sections by Mr. C. G. Chadwin.

Since completion of this paper two other relevant communications have appeared. Currie and Smith (1952), under the title " Multiple primary spontaneous-healing squamous-cell carcinomata of the skin ", describe 2 new cases. They maintain that the lesions they describe are unrelated to molluscum sebaceum. Whittle and Lyell (1952), in commenting on a case of molluscum sebaceum which they demonstrated at the Royal Society of Medicine, " wonder if Dr. Ferguson Smith's cases of multiple self-healing epithelioma, in spite of their familial tendency, are really very different from these kerato-acanthomata ".

### REFERENCES.

AYRES, S.—(1948) *Arch. Derm. Syph., Chicago*, **58**, 584.
BEARE, J. M.—(1953) *Brit. J. Surg.* (in press).
CHARTERIS, A. A.—(1951) *Amer. J. Roentgenol.*, **65**, 459.
CURRIE, A. R., AND SMITH, J. F.—(1952) *J. Path. Bact.*, **44**, 827.
DUNN, J. S., AND SMITH, J.. F.—(1934) *Brit. J. Derm.*, **46**, 519.
GRZYBOWSKI, M.—(1950) *Ibid.*, **62**, 310.
MACCORMAC, H., AND SCARFF, R. W.—(1936) *Ibid.*, **48**, 624.
MUSSO, L. (for GORDON, H.).—(1950) *Proc. R. Soc. Med.*, **43**, 838.
ROOK, A., AND WHIMSTER, I.—(1950) *Arch. belges. Derm.*, **6**, 137.
SMITH, J. F.—(1934) *Brit. J. Derm.*, **46**, 267.—(1948) *Ibid.*, **60**, 315.
SOMMERVILLE, J., AND MILNE, J. A.—(1950) *Ibid.*, **62**, 485.
WHITTLE, C. H., AND LYELL, A.—(1952) *Ibid.*, **64**, 424.
WITTEN, V. H., and ZAK, F. G.—(1952) *Cancer*, **5**, 539.

---

### EXPLANATION OF PLATES.

FIG. 1.—Molluscum sebaceum (Case 1) undergoing spontaneous regression following biopsy. × 2. (*a*) Typical lesion before biopsy. (*b*) 3 weeks after biopsy. (*c*) 11 weeks after biopsy. (*d*) Residual scar 23 weeks after biopsy.

FIG. 2.—Molluscum sebaceum (Case 2). Central vertical section showing the central keratin-filled crypt bounded by hyperplastic epithelium. H. & E. × 7.

FIG. 3.—Molluscum sebaceum (Case 2). Vertical section near periphery of same lesion as Fig. 2 showing secondary crypts and the papillary projections forming their walls. Van Gieson. × 10.

FIG. 4.—Molluscum sebaceum. Elastic fibres engulfed by the advancing margin of hyperplastic epithelium. Verhoeff's elastic. × 90.

FIG. 5.—Molluscum sebaceum (Case 1). Epithelial cell nests penetrating into the dermis, which is infiltrated by chronic inflammatory cells. H. & E. × 55.

Fouracres and Whittick.


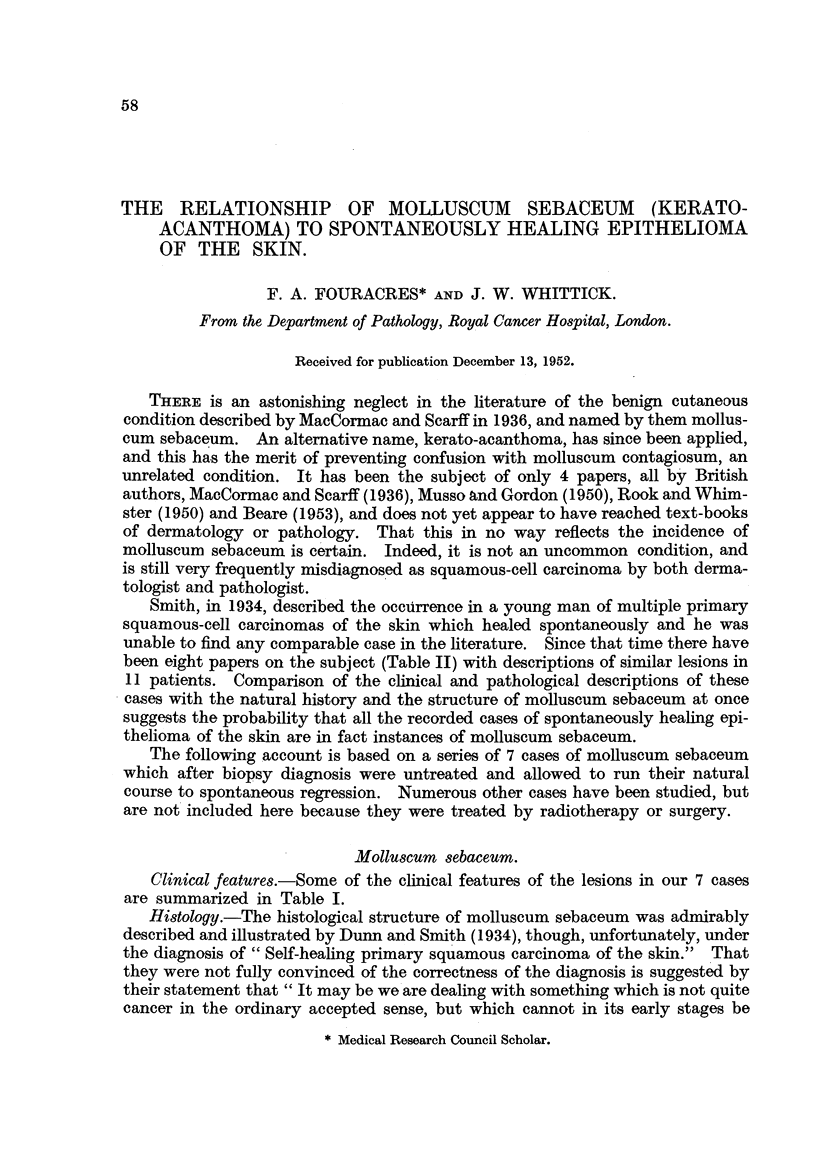

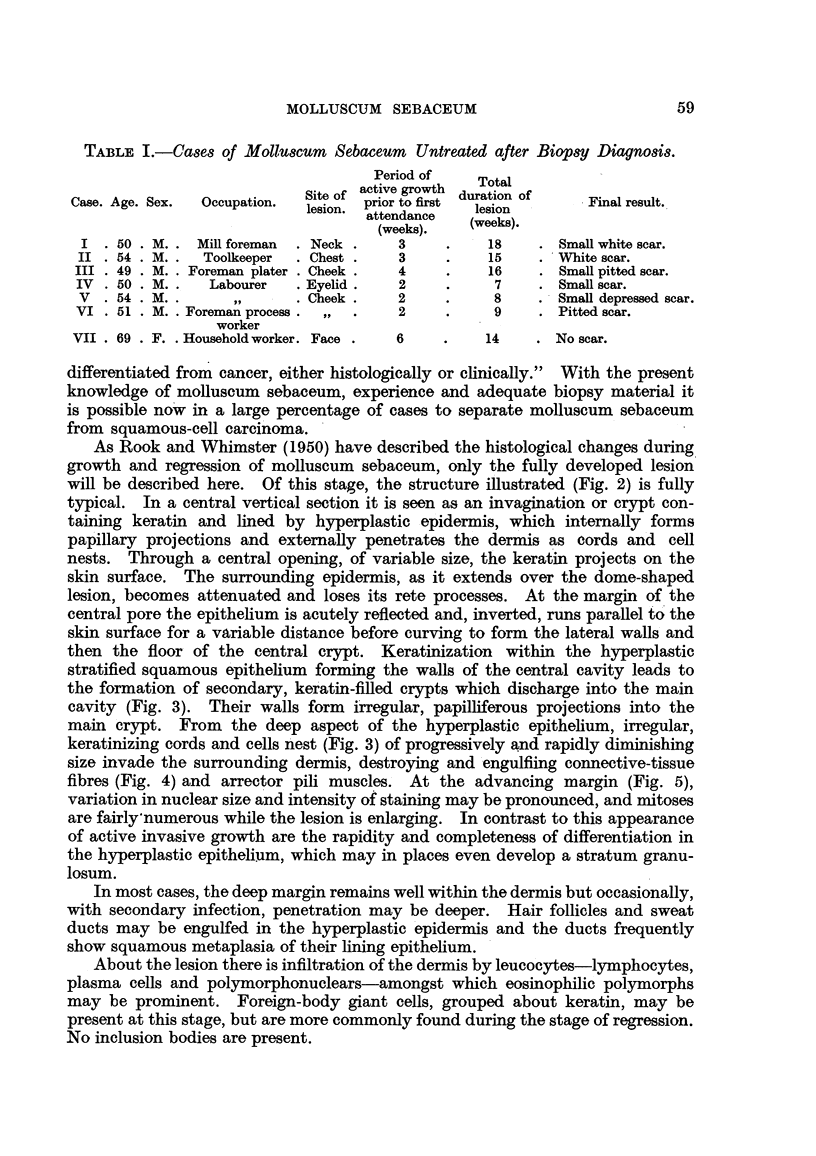

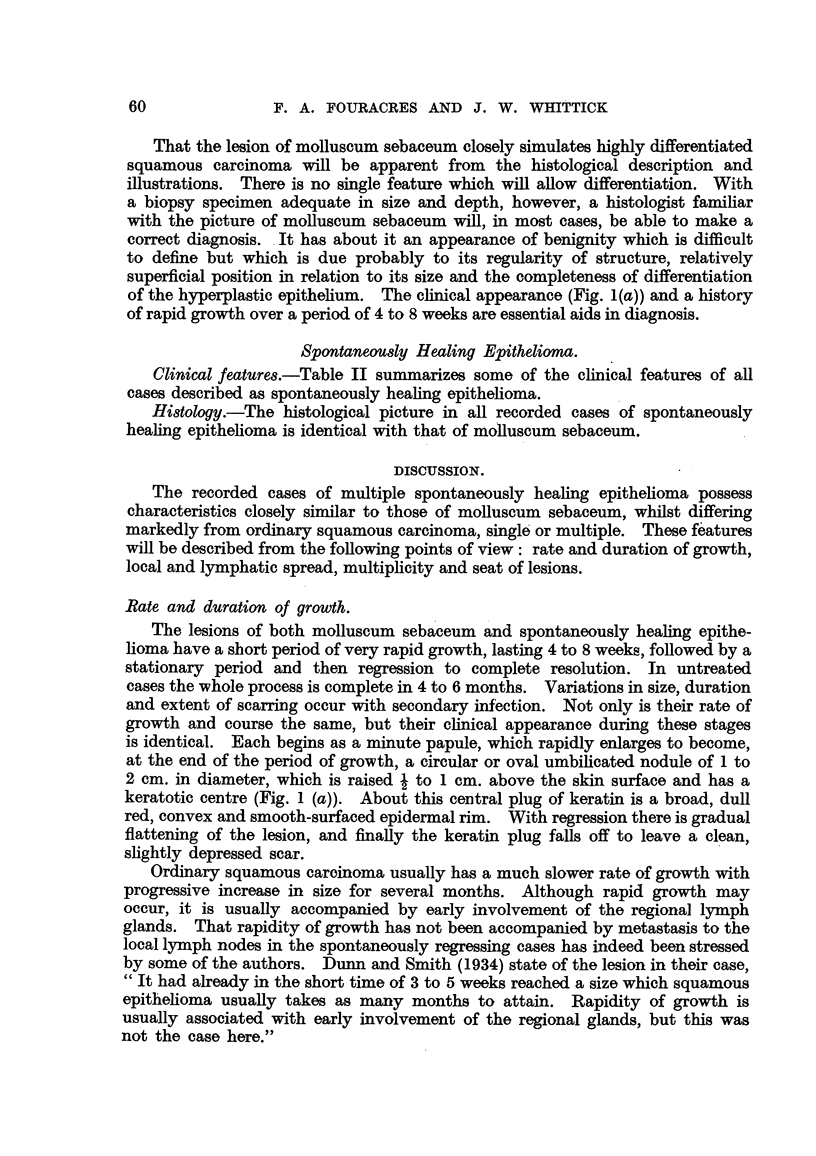

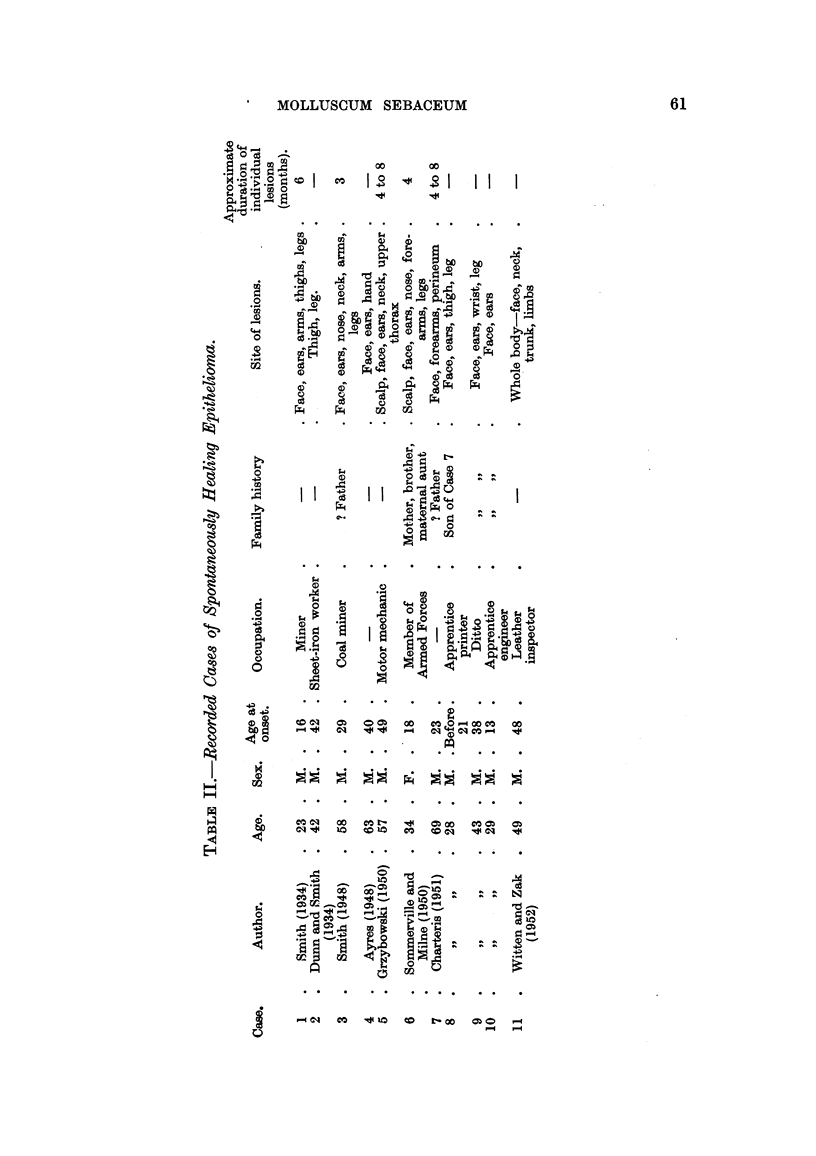

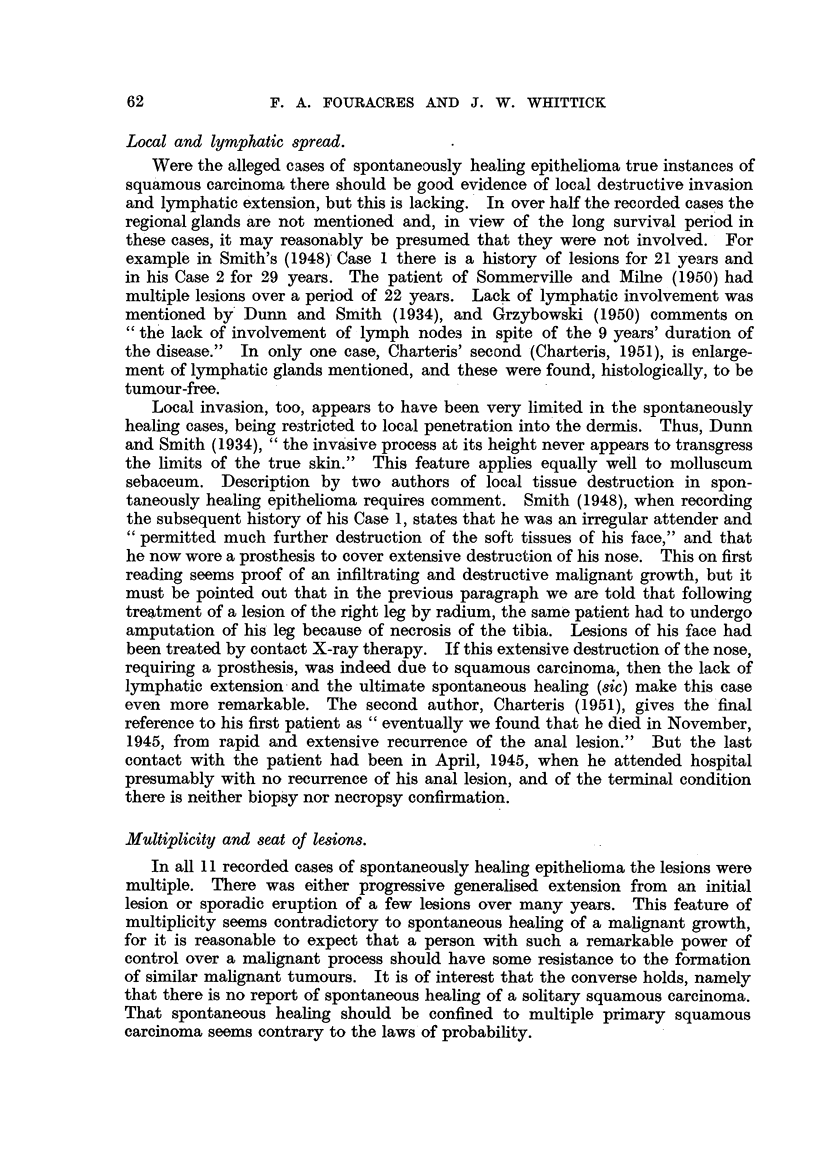

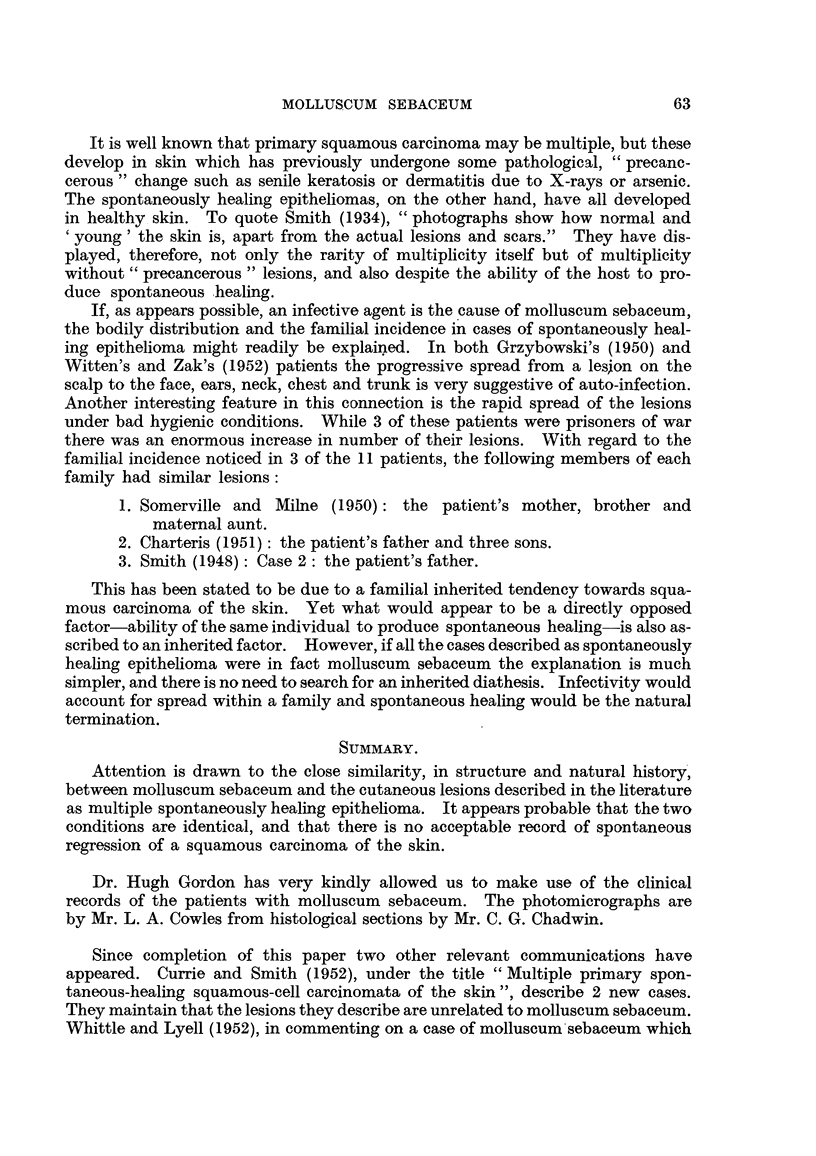

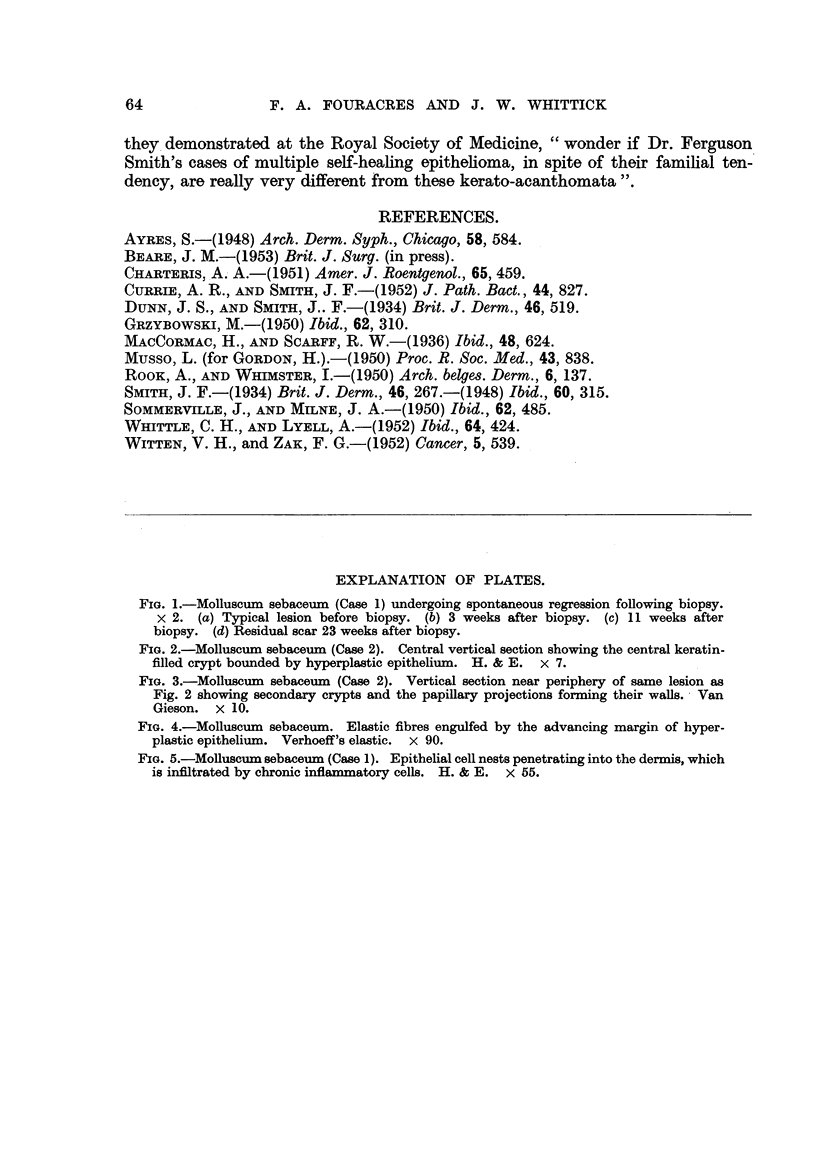

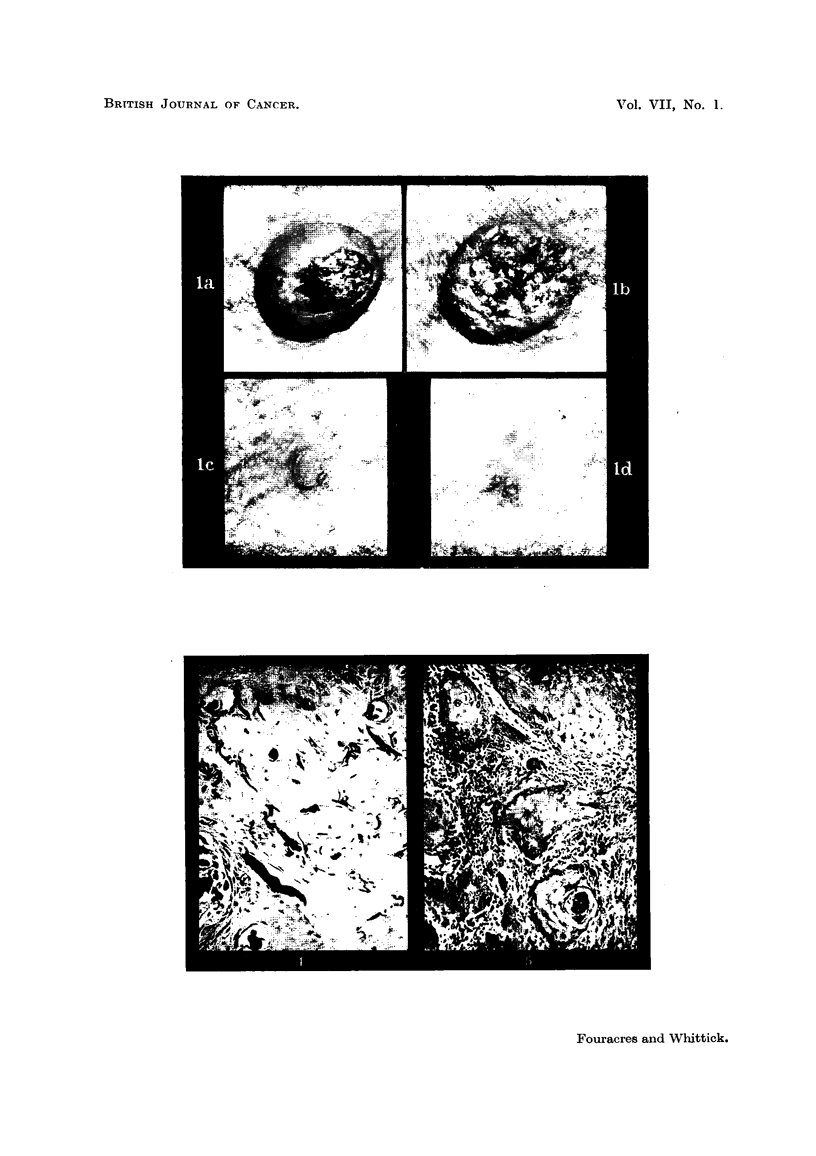

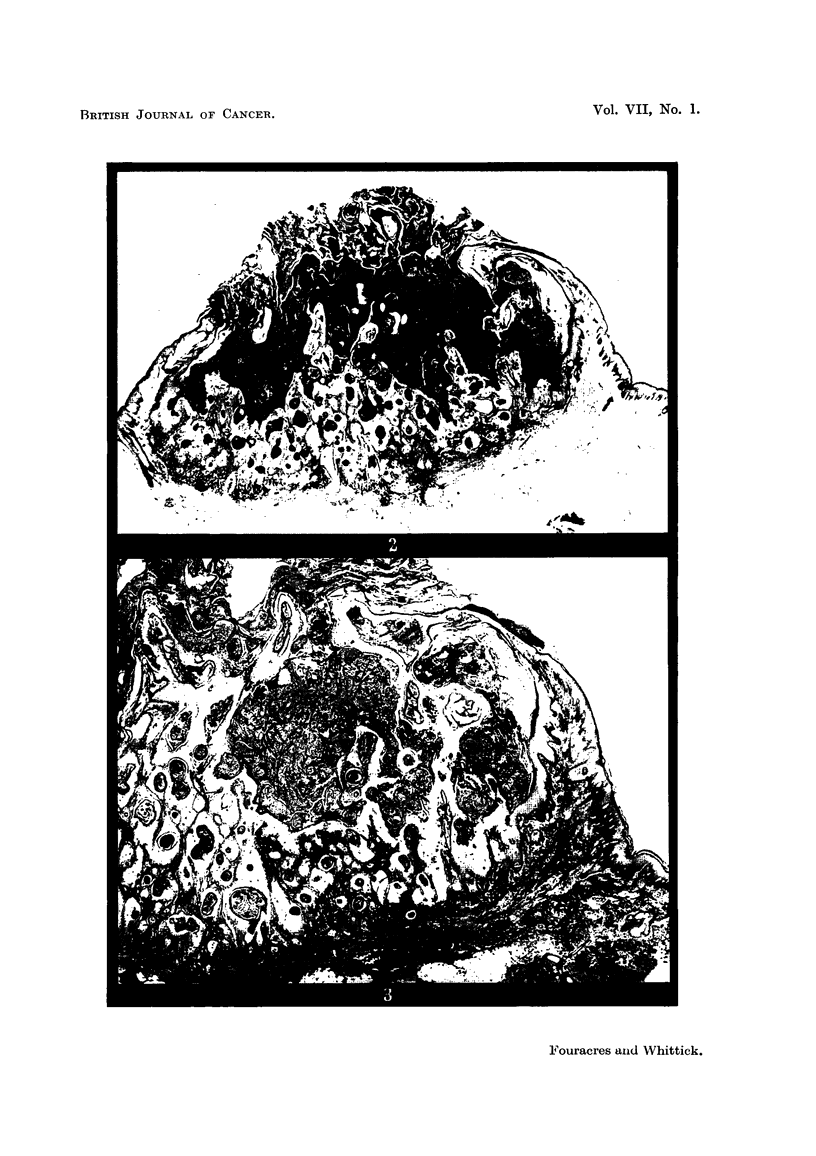


## References

[OCR_00407] CHARTERIS A. A. (1951). Self-healing epithelioma of the skin.. Am J Roentgenol Radium Ther.

[OCR_00337] DARCY D. A. (1952). Survival of adrenal gland homografts in the rabbit's skin.. Nature.

[OCR_00339] Duran-Reynals F. (1942). TISSUE PERMEABILITY AND THE SPREADING FACTORS IN INFECTION : A Contribution to the Host:Parasite Problem.. Bacteriol Rev.

[OCR_00342] FOULDS L. (1949). Mammary tumours in hybrid mice; growth and progression of spontaneous tumours.. Br J Cancer.

[OCR_00416] ROOK A., WHIMSTER I. (1950). Le kératoacanthome.. Arch Belg Dermatol Syphiligr.

[OCR_00420] WHITTLE C. H., LYELL A. (1952). Molluscum sebaceum kerato-acanthoma, benign epithelioma.. Br J Dermatol.

[OCR_00421] WITTEN V. H., ZAK F. G. (1952). Multiple, primary, self-healing prickle-cell epithelioma of the skin.. Cancer.

